# Bioactives and Traditional Herbal Medicine for the Treatment of Cardiovascular/Cerebrovascular Diseases

**DOI:** 10.1155/2014/495323

**Published:** 2014-06-16

**Authors:** Joen-Rong Sheu, Pitchairaj Geraldine, Mao-Hsiung Yen

**Affiliations:** ^1^Graduate Institute of Medical Sciences, College of Medicine, Taipei Medical University, Taipei 110, Taiwan; ^2^Department of Animal Science, Bharathidasan University, Tiruchirappalli, Tamil Nadu 620 024, India; ^3^Department of Pharmacology, National Defense Medical Center, Taipei, Taiwan

The cardiovascular diseases (CVDs) have been the major cause of morbidity and mortality in developed countries over the last several decades, and developing countries are rapidly catching up with this epidemic disease. The underlying pathology is atheromatous vascular disease, resulting in coronary artery disease (CAD), cerebrovascular disease, peripheral vascular disease, and the subsequent development of heart failure and cardiac arrhythmias. There is extensive evidence to show that drug treatment of conventional risk factors is effective in reducing cardiovascular events. More effective treatment of CVD with various classes of antihypertensive drugs has been associated with greater benefits, but some recent studies suggest we may be reaching the optimal level of treated blood pressure in some patient groups. Apart from the treatment of cardiovascular risk factors with pharmacological agents and the use of antithrombotic drugs, there is growing awareness of the role of dietary factors and herbal medicines in the prevention of CVD and the possibility of their use in treatment. In this special issue on bioactives and traditional herbal medicine for the treatment of cardiovascular/cerebrovascular diseases, we called for limited research and review papers on such subjects.

In spite of the major advances of neuroprotective therapeutic approaches for treating ischemic stroke over the last decade, stroke is still a serious problem for which effective drug therapy is not yet available. In the search for neuroprotective agents from natural sources, a number of plant extracts and several natural products were isolated and reported to provide neuroprotection against ischemic stroke. A few papers in this special issue address the neuroprotective effects of Chinese herbal medicine and natural compounds. For instance, Chinese herbal formula Sini Tang (SNT), a decoction that consisted of four herbs:* Aconitum carmichaelii*,* Cinnamomum cassia*,* Zingiber officinale*, and* Glycyrrhiza uralensis,* was reported to improve cardiac function after myocardial infarction (MI) in rats. Similarly, another paper in this issue reports the neuroprotective effect of formula moschus combined with borneolum synthcticum from traditional Chinese medicine on ischemia stroke in rats. In this study it was found that this formula significantly ameliorates neurobehavioral disturbances, shrinks relative infarct size, rescues neural dysfunction, and prevents neuron cells from apoptosis caused by cerebral ischemia or reperfusion to relieve brain damage.

The antiatherosclerotic effect of Guanxinkang (GXK) decoction on the apoptosis, mitochondrial membrane potential (MMP), and endoplasmic reticulum stress (ERS) of human umbilical vein endothelial cells (HUVEC) pretreated with homocysteinemia was presented in this special issue. A review paper in this special issue presents the effects and mechanisms of Chinese herbal medicine in ameliorating myocardial ischemia-reperfusion injury. Moreover, our own paper describes the neuroprotective effect and mechanisms of hinokitiol, a tropolone related compound found in the heartwood cupressaceous plants, in rats against middle cerebral artery occlusion- (MCAO-) induced thromboembolic stroke.

A therapeutic effect of QSYQ, a drug commonly used to treat heart dysfunction in clinical practice in China, has been evaluated against pig myocardial ischemia (MI). This paper addressed the issue that therapeutic QSYQ administration regulates vasoactive factors to improve myocardial oxygen supply, reduce myocardial injury, improve cardiac function, and inhibit myocardial apoptosis via decreasing the level of TNF-*α* and active caspase-3. Subsequently, this special issue described the antihypercholesterolemic and antioxidative properties of an ethanolic extract of* Piper betle *and of its active constituent, eugenol, in experimental hypercholesterolemia in Wistar rats. This paper provides well understanding of the fact that the hypercholesterolemia-ameliorating effect is better defined in eugenol-treated rats than in* Piper betle *extract-treated rats, and it was almost as effective as that of the standard lipid-lowering drug, lovastatin. A paper in this special issue describes the antiplatelet activity of* Morus alba *leaves extract mediated via inhibiting granule secretion and blocking the phosphorylation of extracellular signal-regulated kinase and Akt. Finally, a clinical study about the effect of Ayurveda therapies on the cardiac autonomic dysfunction was offered in this special issue. This is the first study on positive modulation of cardiac autonomic activity after adjuvant Ayurveda treatment in ischemic stroke. We anticipate that this special issue presents innovative knowledge to increase the therapeutic value of herbal and/or Chinese medicines for treatment or prevention of cardiovascular and ischemia-reperfusion injury-related disorders.

*Joen-Rong  Sheu* *Joen-Rong  Sheu* *Pitchairaj  Geraldine* *Pitchairaj  Geraldine* *Mao-Hsiung  Yen* *Mao-Hsiung  Yen*

